# *catena*-Poly[[diaqua­bis(1*H*-imidazole-κ*N* ^3^)cobalt(II)]-μ-2,3,5,6-tetra­chloro­tereph­thal­ato-κ^2^ *O* ^1^:*O* ^4^]

**DOI:** 10.1107/S1600536812005429

**Published:** 2012-03-03

**Authors:** Chang-Ge Zheng, Peng Zhang, Ping Li, Pei-Pei Zhang

**Affiliations:** a School of Chemical and Materials Engineering, Jiangnan University, 1800 Lihu Road, Wuxi, Jiangsu Province 214122, People’s Republic of China

## Abstract

In the title compound, [Co(C_8_Cl_4_O_4_)(C_3_H_4_N_2_)_2_(H_2_O)_2_]_*n*_, the Co^II^ ion displays a distorted octa­hedral coordination geometry with two O atoms from two monodentate tetra­chloro­terephthalate dianions, two N atoms from two imidazole mol­ecules and two O atoms from two water mol­ecules. The Co^II^ ions are connected *via* the tetra­chloro­terephthalate dianions into a chain running along the crystallographic [110] direction. Adjacent chains are linked into a two-dimensional network arranged parallel to (010) by classical N—H⋯O and O—H⋯O hydrogen bonds.

## Related literature

For magnetism, gas storage and electrooptic properties, see: Kumar *et al.* (2009 ▶); Farha *et al.* (2009 ▶); Zhou *et al.* (2006 ▶); Mulder *et al.* (2005 ▶); Zhang *et al.* (2007 ▶). For the geometric parameters of related compounds, see: Murugavel *et al.* (2002 ▶); Rogan *et al.* (2006 ▶); Tong *et al.* (2002 ▶); Zhang & Lu (2004 ▶).
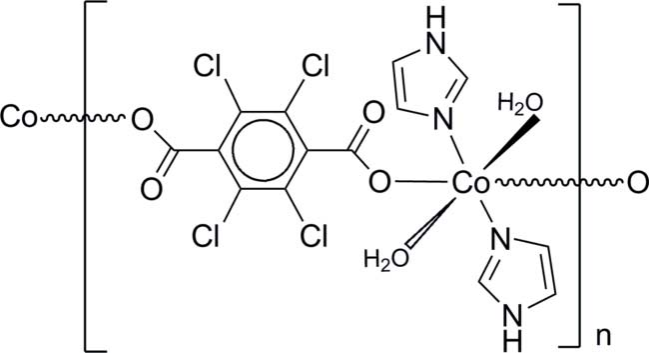


## Experimental

### 

#### Crystal data

[Co(C_8_Cl_4_O_4_)(C_3_H_4_N_2_)_2_(H_2_O)_2_]
*M* *_r_* = 533.01
Monoclinic, 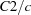

*a* = 18.646 (4) Å
*b* = 12.068 (2) Å
*c* = 10.741 (2) Å
β = 120.76 (3)°
*V* = 2076.9 (9) Å^3^
*Z* = 4
Mo *K*α radiation
μ = 1.38 mm^−1^
*T* = 295 K
0.58 × 0.52 × 0.31 mm

#### Data collection

Bruker SMART APEXII CCD diffractometer
Absorption correction: multi-scan (*SADABS*; Sheldrick, 1996 ▶) *T*_min_ = 0.677, *T*_max_ = 1.000
6189 measured reflections
2342 independent reflections
1959 reflections with *I* > 2σ(*I*)
*R*_int_ = 0.022

#### Refinement

*R*[*F* ^2^ > 2σ(*F* ^2^)] = 0.032
*wR*(*F* ^2^) = 0.078
*S* = 1.06
2342 reflections
133 parameters
H-atom parameters constrained
Δρ_max_ = 0.27 e Å^−3^
Δρ_min_ = −0.29 e Å^−3^

### 

Data collection: *APEX2* (Bruker, 2005 ▶); cell refinement: *SAINT* (Bruker, 2005 ▶); data reduction: *SAINT*; program(s) used to solve structure: *SHELXS97* (Sheldrick, 2008 ▶); program(s) used to refine structure: *SHELXL97* (Sheldrick, 2008 ▶); molecular graphics: *PLATON* (Spek, 2009 ▶); software used to prepare material for publication: *SHELXL97*.

## Supplementary Material

Crystal structure: contains datablock(s) global, I. DOI: 10.1107/S1600536812005429/rk2332sup1.cif

Structure factors: contains datablock(s) I. DOI: 10.1107/S1600536812005429/rk2332Isup2.hkl

Additional supplementary materials:  crystallographic information; 3D view; checkCIF report

## Figures and Tables

**Table 1:** Hydrogen-bond geometry (Å, °)

*D*—H⋯*A*	*D*—H	H⋯*A*	*D*⋯*A*	*D*—H⋯*A*
O3—H3*A*⋯O1^i^	0.85	1.94	2.7681 (19)	166
O3—H3*B*⋯O2^ii^	0.85	2.01	2.696 (2)	137
N2—H2*B*⋯O2^iii^	0.86	1.96	2.803 (2)	167

Symmetry codes: (i) 
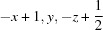
; (ii) 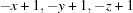; (iii) 
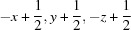
.

**Fig. 1.**
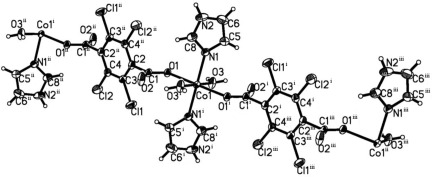
ORTEP plot of a fragment of the title compound (with the atom numbering scheme) showing the coordination environment of Co1 atom and the one–dimensional polymeric structure. Displacement ellipsoids are drawn at the 50% probability level. Symmetry codes: (i) 1-x, y, 1/2-z; (ii) 1-x, 1-y, 1-z; (iii) 1/2-x, 1/2+y, 1/2-z.

## supplementary crystallographic information

### Comment

The design and synthesis of coordination polymers has attracted great interest in functional solid–state materials, owing to their excellent properties in magnetism, gas storage and electrooptic materials (Kumar *et al.*, 2009; Farha *et al.*, 2009; Zhou *et al.*, 2006). Herein, compared with 1,4–benzenedicarboxylic acid, tetrachloroterephthalic acid can be used to construct materials which have different properties. Computational study suggests that 1,4–benzenedicarboxylic acid with chemical modification have better adsorption property in gas storage (Mulder *et al.*, 2005; Zhang *et al.*, 2007).

Single–crystal X–ray structural analysis reveals that the title cobalt(II) complex in crystal built from one–dimensional linear chains running along the crystallographic direction [1 1 0]. As shown in Fig. 1, the coordination geometry around the Co(II) atom is a slightly distorted octahedron with N_2_O_4_ binding set. In the octahedron unit, two O atoms from the tetrachloroterephthalate dianions ligands and two N atoms from the imidazole molecules form the equatorial plane and the axial position is occupied by O atoms from two water molecules. The Co—O bond lengths are 2.1653 (14)Å and 2.0865 (14)Å and agree well with the reported (Murugavel *et al.*, 2002; Rogan *et al.*, 2006). The Co—N bond length are 2.0896 (17)Å, which are comparable with the reported values in the similar complexes (Tong *et al.*, 2002; Zhang & Lu, 2004). In addition, the imidazole and water molecules act as donors in N—H···O and O—H···O hydrogen bonds (Table 1). Adjacent one–dimensional chains are linked into a two–dimensional network arranged along the crystallographic *b* axis by classical N2—H2B···O2^iii^ and O3—H3A···O1^i^ hydrogen bonds with the angles of 167° and 166°, respectivly. Symmetry codes: (i) -*x*+1, *y*, -*z*+1/2; (iii) -*x*+1/2, *y*+1/2, -*z*+1/2.

### Experimental

All the reagents and solvents employed were commercially available. Tetrachloroterephthalic acid was purified by recrystallization. In a 15 cm long tube, a solution of sodium tetrachloroterephthalate (0.0346 g, 0.1 mmol) and imidazol (0.0068 g, 0.1 mmol) in 5 mL methanol was carefully layered on top of a bilayer solution comprised of a solution of Co(NO_3_)_2_×6H_2_O (0.0291 g, 0.10 mmol) in 5 mL water on the bottom and a buffer solvent of 6 mL ethyl acetate on the top at room temperature. Half a month later, pink block–shaped crystals were obtained, washed with water, and dried on air (0.0618 g, yield: 58% based on Co). Elemental analysis(%) calcd. for C_14_H_12_Cl_4_CoN_4_O_6_: C, 31.52; H, 2.25; N, 10.51. Found: C, 31.37; H, 2.24; N, 10.47%.

### Refinement

All the other H atoms were placed in geometrically idealized positions and constrained to ride on their parent atoms with C—H, O—H and N—H distances of 0.93Å, 0.85Å and 0.86Å with *U*_iso_(H) = 1.2(1.5)*U*_eq_(C, O, N). All C—Cl bond lengths were restrained to 1.728–1.729 (2)Å.

### Crystal data

**Table:**

[Co(C_8_Cl_4_O_4_)(C_3_H_4_N_2_)_2_(H_2_O)_2_]	*Z* = 4
*M**_r_* = 533.01	*F*(000) = 1068
Monoclinic, *C*2/*c*	*D*_x_ = 1.705 Mg m^−^^3^
Hall symbol: -C 2yc	Mo *K*α radiation, λ = 0.71073 Å
*a* = 18.646 (4) Å	µ = 1.38 mm^−^^1^
*b* = 12.068 (2) Å	*T* = 295 K
*c* = 10.741 (2) Å	Block, pink
β = 120.76 (3)°	0.58 × 0.52 × 0.31 mm
*V* = 2076.9 (9) Å^3^	

### Data collection

**Table:**

Bruker SMART APEXII CCD diffractometer	2342 independent reflections
Radiation source: fine-focus sealed tube	1959 reflections with *I* > 2σ(*I*)
Graphite monochromator	*R*_int_ = 0.022
φ and ω scans	θ_max_ = 27.5°, θ_min_ = 3.4°
Absorption correction: multi-scan (*SADABS*; Sheldrick, 1996)	*h* = −18→24
*T*_min_ = 0.677, *T*_max_ = 1.000	*k* = −12→15
6189 measured reflections	*l* = −13→11

### Refinement

**Table:**

Refinement on *F*^2^	Primary atom site location: structure-invariant direct methods
Least-squares matrix: full	Secondary atom site location: difference Fourier map
*R*[*F*^2^ > 2σ(*F*^2^)] = 0.032	Hydrogen site location: inferred from neighbouring sites
*wR*(*F*^2^) = 0.078	H-atom parameters constrained
*S* = 1.06	*w* = 1/[σ^2^(*F*_o_^2^) + (0.041*P*)^2^ + 0.2172*P*] where *P* = (*F*_o_^2^ + 2*F*_c_^2^)/3
2342 reflections	(Δ/σ)_max_ < 0.001
133 parameters	Δρ_max_ = 0.27 e Å^−^^3^
0 restraints	Δρ_min_ = −0.29 e Å^−^^3^

### Special details

**Table:**

Geometry. All s.u.'s (except the s.u. in the dihedral angle between two l.s. planes) are estimated using the full covariance matrix. The cell s.u.'s are taken into account individually in the estimation of s.u.'s in distances, angles and torsion angles; correlations between s.u.'s in cell parameters are only used when they are defined by crystal symmetry. An approximate (isotropic) treatment of cell s.u.'s is used for estimating s.u.'s involving l.s. planes.
Refinement. Refinement of *F*^2^ against ALL reflections. The weighted *R*–factor *wR* and goodness of fit *S* are based on *F*^2^, conventional *R*–factors *R* are based on *F*, with *F* set to zero for negative *F*^2^. The threshold expression of *F*^2^ > σ(*F*^2^) is used only for calculating *R*–factors(gt) *etc*. and is not relevant to the choice of reflections for refinement. *R*–factors based on *F*^2^ are statistically about twice as large as those based on *F*, and *R*–factors based on ALL data will be even larger.

### Fractional atomic coordinates and isotropic or equivalent isotropic displacement parameters (Å^2^)

**Table:**

	*x*	*y*	*z*	*U*_iso_*/*U*_eq_	
Co1	0.5000	0.5000	0.5000	0.01850 (11)	
Cl1	0.40036 (3)	0.13509 (5)	0.26222 (6)	0.04701 (19)	
Cl2	0.29749 (4)	0.00958 (5)	−0.03453 (6)	0.04412 (17)	
O1	0.40828 (7)	0.41632 (11)	0.30419 (12)	0.0236 (3)	
O2	0.31637 (9)	0.35243 (14)	0.36131 (15)	0.0414 (4)	
O3	0.58228 (8)	0.50886 (12)	0.42281 (15)	0.0323 (4)	
H3A	0.5784	0.4877	0.3441	0.048*	
H3B	0.6151	0.5642	0.4520	0.048*	
N1	0.44543 (10)	0.65072 (14)	0.40112 (17)	0.0268 (4)	
C1	0.34446 (11)	0.36395 (17)	0.27926 (18)	0.0229 (4)	
C2	0.29635 (11)	0.30562 (17)	0.13275 (19)	0.0234 (4)	
C3	0.31676 (11)	0.19887 (17)	0.11602 (19)	0.0263 (4)	
C4	0.27131 (11)	0.14324 (17)	−0.0150 (2)	0.0251 (4)	
C5	0.48095 (13)	0.7461 (2)	0.3896 (3)	0.0399 (6)	
H5A	0.5380	0.7575	0.4302	0.048*	
C6	0.42110 (16)	0.8213 (2)	0.3105 (3)	0.0496 (6)	
H6A	0.4289	0.8929	0.2873	0.060*	
N2	0.34743 (11)	0.77162 (18)	0.27174 (19)	0.0414 (5)	
H2B	0.2986	0.8004	0.2202	0.050*	
C8	0.36442 (13)	0.6702 (2)	0.3279 (2)	0.0365 (5)	
H8A	0.3242	0.6193	0.3170	0.044*	

### Atomic displacement parameters (Å^2^)

**Table:**

	*U*^11^	*U*^22^	*U*^33^	*U*^12^	*U*^13^	*U*^23^
Co1	0.01555 (17)	0.0220 (2)	0.01592 (18)	−0.00026 (13)	0.00661 (14)	−0.00168 (13)
Cl1	0.0432 (3)	0.0393 (4)	0.0280 (3)	0.0089 (3)	−0.0038 (2)	−0.0019 (2)
Cl2	0.0459 (3)	0.0311 (3)	0.0362 (3)	0.0067 (2)	0.0072 (3)	−0.0097 (2)
O1	0.0224 (6)	0.0281 (8)	0.0176 (6)	−0.0059 (5)	0.0083 (5)	−0.0036 (5)
O2	0.0381 (8)	0.0619 (12)	0.0288 (7)	−0.0277 (8)	0.0205 (7)	−0.0187 (7)
O3	0.0289 (7)	0.0455 (10)	0.0293 (7)	−0.0142 (6)	0.0197 (6)	−0.0163 (6)
N1	0.0234 (8)	0.0259 (10)	0.0250 (8)	0.0028 (7)	0.0080 (7)	−0.0005 (7)
C1	0.0210 (9)	0.0259 (11)	0.0168 (8)	−0.0047 (7)	0.0061 (7)	−0.0045 (8)
C2	0.0218 (9)	0.0279 (12)	0.0198 (9)	−0.0065 (8)	0.0100 (7)	−0.0037 (8)
C3	0.0218 (9)	0.0300 (12)	0.0197 (9)	−0.0023 (8)	0.0052 (7)	0.0000 (8)
C4	0.0258 (9)	0.0213 (11)	0.0250 (9)	−0.0028 (8)	0.0108 (8)	−0.0048 (8)
C5	0.0316 (11)	0.0337 (14)	0.0441 (13)	0.0009 (10)	0.0118 (10)	0.0090 (11)
C6	0.0520 (15)	0.0357 (15)	0.0581 (16)	0.0068 (12)	0.0260 (13)	0.0156 (12)
N2	0.0379 (10)	0.0451 (13)	0.0390 (10)	0.0214 (9)	0.0179 (9)	0.0111 (9)
C8	0.0286 (10)	0.0378 (14)	0.0400 (12)	0.0074 (9)	0.0153 (10)	0.0035 (10)

### Geometric parameters (Å, º)

**Table:**

Co1—O3	2.0865 (14)	N1—C5	1.365 (3)
Co1—O3^i^	2.0865 (14)	C1—C2	1.527 (2)
Co1—N1	2.0896 (17)	C2—C3	1.381 (3)
Co1—N1^i^	2.0896 (17)	C2—C4^ii^	1.393 (3)
Co1—O1	2.1653 (14)	C3—C4	1.389 (3)
Co1—O1^i^	2.1653 (14)	C4—C2^ii^	1.393 (3)
Cl1—C3	1.728 (2)	C5—C6	1.350 (3)
Cl2—C4	1.729 (2)	C5—H5A	0.9300
O1—C1	1.250 (2)	C6—N2	1.355 (3)
O2—C1	1.242 (2)	C6—H6A	0.9300
O3—H3A	0.8500	N2—C8	1.329 (3)
O3—H3B	0.8500	N2—H2B	0.8600
N1—C8	1.319 (3)	C8—H8A	0.9300
			
O3—Co1—O3^i^	180.0	O2—C1—C2	116.16 (16)
O3—Co1—N1	91.11 (6)	O1—C1—C2	116.56 (16)
O3^i^—Co1—N1	88.89 (6)	C3—C2—C4^ii^	118.49 (17)
O3—Co1—N1^i^	88.89 (6)	C3—C2—C1	120.55 (16)
O3^i^—Co1—N1^i^	91.11 (6)	C4^ii^—C2—C1	120.89 (18)
N1—Co1—N1^i^	180.0	C2—C3—C4	121.12 (17)
O3—Co1—O1	90.80 (5)	C2—C3—Cl1	118.56 (14)
O3^i^—Co1—O1	89.20 (5)	C4—C3—Cl1	120.32 (16)
N1—Co1—O1	88.56 (6)	C3—C4—C2^ii^	120.39 (18)
N1^i^—Co1—O1	91.44 (6)	C3—C4—Cl2	120.67 (15)
O3—Co1—O1^i^	89.20 (5)	C2^ii^—C4—Cl2	118.94 (14)
O3^i^—Co1—O1^i^	90.80 (5)	C6—C5—N1	109.9 (2)
N1—Co1—O1^i^	91.44 (6)	C6—C5—H5A	125.0
N1^i^—Co1—O1^i^	88.56 (6)	N1—C5—H5A	125.0
O1—Co1—O1^i^	180.0	C5—C6—N2	106.1 (2)
C1—O1—Co1	129.54 (11)	C5—C6—H6A	126.9
Co1—O3—H3A	132.4	N2—C6—H6A	126.9
Co1—O3—H3B	115.5	C8—N2—C6	107.42 (19)
H3A—O3—H3B	106.4	C8—N2—H2B	126.3
C8—N1—C5	104.86 (19)	C6—N2—H2B	126.3
C8—N1—Co1	124.72 (16)	N1—C8—N2	111.7 (2)
C5—N1—Co1	130.35 (14)	N1—C8—H8A	124.2
O2—C1—O1	127.27 (16)	N2—C8—H8A	124.2
			
O3—Co1—O1—C1	165.06 (16)	O1—C1—C2—C4^ii^	−94.6 (2)
O3^i^—Co1—O1—C1	−14.94 (16)	C4^ii^—C2—C3—C4	0.1 (3)
N1—Co1—O1—C1	−103.85 (17)	C1—C2—C3—C4	177.30 (18)
N1^i^—Co1—O1—C1	76.15 (17)	C4^ii^—C2—C3—Cl1	−179.71 (14)
O3—Co1—N1—C8	136.25 (17)	C1—C2—C3—Cl1	−2.5 (3)
O3^i^—Co1—N1—C8	−43.75 (17)	C2—C3—C4—C2^ii^	−0.1 (3)
O1—Co1—N1—C8	45.49 (17)	Cl1—C3—C4—C2^ii^	179.71 (15)
O1^i^—Co1—N1—C8	−134.51 (17)	C2—C3—C4—Cl2	−179.99 (15)
O3—Co1—N1—C5	−40.3 (2)	Cl1—C3—C4—Cl2	−0.1 (3)
O3^i^—Co1—N1—C5	139.7 (2)	C8—N1—C5—C6	0.2 (3)
O1—Co1—N1—C5	−131.0 (2)	Co1—N1—C5—C6	177.28 (17)
O1^i^—Co1—N1—C5	49.0 (2)	N1—C5—C6—N2	−0.4 (3)
Co1—O1—C1—O2	3.9 (3)	C5—C6—N2—C8	0.4 (3)
Co1—O1—C1—C2	−174.82 (12)	C5—N1—C8—N2	0.0 (3)
O2—C1—C2—C3	−90.6 (2)	Co1—N1—C8—N2	−177.26 (14)
O1—C1—C2—C3	88.3 (2)	C6—N2—C8—N1	−0.2 (3)
O2—C1—C2—C4^ii^	86.5 (2)		

Symmetry codes: (i) −*x*+1, −*y*+1, −*z*+1; (ii) −*x*+1/2, −*y*+1/2, −*z*.

### Hydrogen-bond geometry (Å, º)

**Table:**

*D*—H···*A*	*D*—H	H···*A*	*D*···*A*	*D*—H···*A*
O3—H3*A*···O1^iii^	0.85	1.94	2.7681 (19)	166
O3—H3*B*···O2^i^	0.85	2.01	2.696 (2)	137
N2—H2*B*···O2^iv^	0.86	1.96	2.803 (2)	167

Symmetry codes: (i) −*x*+1, −*y*+1, −*z*+1; (iii) −*x*+1, *y*, −*z*+1/2; (iv) −*x*+1/2, *y*+1/2, −*z*+1/2.

## References

[bb1] Bruker (2005). *APEX2* and *SAINT* Bruker AXS Inc., Madison, Wisconsin, USA.

[bb2] Farha, O. K., Spokoyny, A. Y. M., Mulfort, K. L., Galli, S., Hupp, J. T. & Mirkin, C. A. (2009). *Small*, **5**, 1727–1731. 10.1002/smll.200900085 19367598

[bb3] Kumar, A., Mayer–Figge, H., Sheldrick, W. S. & Singh, N. (2009). *Eur. J. Inorg. Chem.* pp. 2720–2725.

[bb4] Mulder, F. M., Dingemans, T. J., Wagemake, M. & Kearley, G. J. (2005). *Chem. Phys.* **317**, 113–118.

[bb5] Murugavel, R., Krishnamurthy, D. & Sathiyendiran, M. (2002). *J. Chem. Soc. Dalton Trans.* pp. 34–39.

[bb6] Rogan, J., Poleti, D. & Karanović, L. (2006). *Z. Anorg. Allg. Chem.* **632**, 133–139.

[bb7] Sheldrick, G. M. (1996). *SADABS* University of Göttingen, Germany.

[bb8] Sheldrick, G. M. (2008). *Acta Cryst.* A**64**, 112–122. 10.1107/S0108767307043930 18156677

[bb9] Spek, A. L. (2009). *Acta Cryst.* D**65**, 148–155. 10.1107/S090744490804362X PMC2631630 19171970

[bb10] Tong, M.-L., Li, W., Chen, X.-M. & Ng, S. W. (2002). *Acta Cryst.* E**58**, m186–m188.

[bb11] Zhang, Q.-Z. & Lu, C.-Z. (2004). *Acta Cryst.* E**60**, m1289–m1290.

[bb12] Zhang, L., Wang, Q. & Liu, Y. C. (2007). *J. Phys. Chem. B*, **111**, 4291–4295. 10.1021/jp0713918 17417903

[bb13] Zhou, Y. F., Hong, M. C. & Wu, X. T. (2006). *Chem. Commun.* pp. 135–143. 10.1039/b509458p 16372085

